# Variations of emotion dysregulation in borderline personality disorder: a latent profile analysis approach with adult psychiatric inpatients

**DOI:** 10.1186/s40479-017-0068-2

**Published:** 2017-08-24

**Authors:** Katrina A. Rufino, Thomas E. Ellis, Joshua Clapp, Catherine Pearte, J. Christopher Fowler

**Affiliations:** 1 University of Houston – Downtown and The Menninger Clinic, 1 Main Street, Houston, TX 77002 USA; 2 The Menninger Clinic and Baylor College of Medicine, Houston, TX USA; 3 University of Wyoming, Laramie, WY USA; 4 Stephen F. Austin State University, Nacogdoches, TX USA

**Keywords:** Borderline personality disorder, Latent profile analysis, Inpatient

## Abstract

**Background:**

The purpose of the present study was to identify variations in emotional dysregulation patterns among adults diagnosed with borderline personality disorder (BPD), with an eye toward implications for treatment.

**Methods:**

Latent profile analysis (LPA) was utilized to classify 156 inpatients with BPD, based on patterns of Difficulties in Emotion Regulation Scale (DERS; Gratz and Roemer, J Psychopathol Behav Assess 26: 41-54, 2004) subscale scores.

**Results:**

Results revealed that a three class solution best fit the sample (Low Impairment, Global Dysregulation, and Emotionally Aware). Further analysis of the classes at admission revealed that the Global Dysregulation group reported significantly higher suicidal ideation than either the Low Impairment or Emotionally Aware groups, and that the Global Dysregulation group reported significantly higher functional impairment than the Low Impairment group.

**Conclusions:**

All three groups improved greatly over the course of hospital treatment, although they remained distinguishable at discharge, retaining their positions symptomatically relative to one another. Limitations, implications, and future directions are discussed.

**Electronic supplementary material:**

The online version of this article (doi:10.1186/s40479-017-0068-2) contains supplementary material, which is available to authorized users.

## Background

Borderline personality disorder (BPD) has a prevalence of 10% in psychiatric outpatients and between 15 and 25% of inpatients [24]. The disorder is associated with high comorbididity [44], extensive treatment utilization [5], suicide, and severe functional impairment [33, 36], as well as high costs to society [49]. Despite being the most extensively studied personality disorder [14, 22, 29], the diagnostic and statistical manual (DSM) diagnosis itself is problematic due to the polythetic diagnostic system requiring five out of nine criteria to make a categorical BPD diagnosis. With over 250 possible variants of BPD, significant heterogeneity within the disorder increases the complexity and reduces the accuracy of diagnosing BPD [34]. The clearest examples of inaccuracy come from the American Psychiatric Association’s DSM 5 field trial, which yielded “questionable” inter-rater reliability (kappa = .34) for BPD [38]. The current study addressed a central element of the heterogeneity problem by exploring variations in emotion dysregulation among a large cohort of adults diagnosed with BPD.

The latest iteration of DSM 5 retained the broad criteria definition of BPD as characterized by a “pervasive pattern of instability of interpersonal relationships, self-image, and *affects*, and marked impulsivity…” ([2], p. 663, emphasis added). Previous research by Clifton and Pilkonis [8] demonstrated the importance of affect in BPD, as affective instability had the largest factor loading in a confirmatory factor analysis of BPD criteria, and was the trait with the greatest probability given membership in the BPD class. Furthermore, the negative impact of emotion dysregulation is well-documented [18, 21, 25].

BPD, more generally, is strongly associated with impaired psychosocial functioning, personal economic loss, and increased mortality. Studies have shown that adults with BPD are three times more likely to receive social security disability income than those with other personality disorders, and that more than 60% of adults with BPD received disability income at some point over a 10 year follow up [50]. This comports with Zimmerman and colleagues who reported that depressed patients with BPD were significantly more likely to be chronically unemployed than depressed patients without BPD [52]. Up to 84% of patients with BPD engage in suicidal behavior [45], and up to 10% of those diagnosed with BPD die by suicide [2]. Furthermore, suicide attempts are four to six times more frequent than completed suicides, and are made earlier in life by adults with BPD when compared to depressed adults [45].

Efforts to address reliability, validity, and heterogeneity issues with BPD diagnosis have produced variable results. These include efforts to identify common clusters of DSM BPD diagnostic criteria via factor analysis [4, 6, 23, 47], as well as identifying clusters (subtypes) of patients utilizing latent class analysis [8, 20, 43] based on combinations of BPD criteria [23] or number of criteria [43]. Proposed subtyping has also been proffered based on clinical experience and literature reviews [34, 44]. Generally speaking, inconsistent findings have resulted in a lack of consensus regarding symptom clusters or BPD subtypes.

An alternative approach to understanding borderline psychopathology may be to focus on core features of BPD other than the specific DSM criteria, notably, emotion dysregulation (see, for example, [40]). Selby and Joiner [41] proposed the Emotional Cascade Model, which posits that individuals with BPD experience extreme vacillations in negative emotion induced by rumination on negative affect. The result of this emotional cascade is an intolerable emotional state that leads to dysregulated behaviors that are utilized to distract attention from the negative thoughts to the physical sensations of the dysregulated behaviors [41]. Recent research exploring the model revealed a three-way interaction between elevated BPD symptoms, elevated rumination, and negative emotions, which prospectively predicted dysregulated behavior within the next two-to-three hours. Examples of dysregulated behaviors included non-suicidal self-injury, alcohol use, physical fights, binge eating, and reckless driving [42].

Clinical and empirical data indicate that BPD diagnosis varies in the degree of emotion dysregulation, leading BPD experts [35, 51] to propose typologies (including an affective subtype) that lead to different treatment algorithms [35]. Identifying specific facets of emotion dysregulation (lack of emotional awareness, nonacceptance of emotions, lack of adaptive strategies for coping with strong emotions, and/or impulse control difficulties), may help clinicians personalize treatment plans and reduce iatrogenic effects [12] including adverse polypharmacy [28].

In the present study, we utilized latent profile analysis (LPA) to identify variations in patterns of emotional dysregulation within a BPD sample, based on patterns of subscale scores in the Difficulties in Emotion Regulation Scale (DERS; [19]). The current study differs from earlier classification studies in that we sought to identify varieties of emotional dysregulation within the BPD diagnosis, rather than subgroups of individuals with BPD. Furthermore, participants were sampled from an extended stay inpatient hospital, offering a new perspective over the course of treatment. In addition to identifying varieties of emotion dysregulation patterns in BPD, we also sought to determine how individuals exhibiting such patterns might differ on baseline symptom and functional severity, as well as symptomatology at discharge. Emotion dysregulation in particular was chosen due to the previously established relationship between affective instability and borderline personality disorder [8, 35, 51]. Similarly, well-being and suicidal ideation were chosen for comparison due to their association with BPD in the literature [45, 50, 52].

## Method

### Treatment and participants

The present study was conducted at a private psychiatric hospital, where treatment consists of intensive, multi-modal interventions that include individual, and group psychotherapy, family interventions, addictions services, medication management, 24-h nursing care, psycho-educational groups, and structured interpersonal and recreational activities. The treatment model was prominently influenced by mentalization-based therapy ([1, 3]), together with available training in skills derived from Dialectical Behavior Therapy (DBT; [26]) and other cognitive-behavioral approaches. Interventions are employed in the context of a therapeutic milieu to maximize social engagement, peer support, and learning. Delivery of multimodal interventions is intensive, with an average of 59.4 h of available programming per week. The average length of stay for the present sample was 51.77 days.

Patients at this facility typically suffer from multiple mood, anxiety, alcohol and substance use and personality disorders. Over 99% have histories of unsatisfactory outcomes from multiple previous interventions, including brief and extended hospitalizations. The present study included a convenience sample of 156 individuals with a diagnosis of BPD, ranging in age from 18 to 70 (*M* = 29.43, *SD* = 11.48). A majority of participants (61.8%) was female, and a large majority (88.5%) self-identified as White. The modal participant (45.2%) reported his or her highest level of education as “some college;” 12.1% a reported a high school education, 19.1% a Bachelor’s degree, and 14.6% a graduate degree. Approximately 80% of patients reported suicidal ideation and/or suicidal behavior at admission.

### Procedure

This study was approved by the Institutional Review Board of Baylor College of Medicine. Psychiatric disorders including personality diagnoses were assessed using research versions of the Structured Clinical Interview for DSM-IV Disorders (SCID-I/II). The SCID-I [10, 11] and SCID II (First et al. 2002) were administered by master’s level researchers. Additionally, all patients completed an assessment protocol and a patient information questionnaire that includes demographic information as part of a standard outcomes assessment battery. Domains assessed include depression, anxiety, sleep, personality, attachment, suicidal ideation and behavior, and well-being. Patients electing to participate completed study instruments via an existing computer-based assessment system, with the aid of a research assistant.

### Measures

The *Difficulties in Emotion Regulation Scale* (DERS; [19]) is a self-report measure assessing emotion regulation and dysregulation. It contains 36 items that load onto six factors: Nonacceptance of Emotional Responses (difficulty accepting distress or negative secondary emotional responses), Difficulties in Engaging in Goal-Directed Behavior (difficulty with concentration or accomplishing tasks), Impulse Control Difficulties (difficulty maintaining control of emotional responses), Lack of Emotional Awareness (difficulty recognizing emotions), Limited Access to Emotion Regulation Strategies (difficulty understanding or believing that emotions can be regulated once upset), and Lack of Emotional Clarity (the degree to which an individual understands the emotions they are feeling) [19]. The instrument developers report high internal consistency and strong predictive validity [19]. Normative data for individual subscales are available for both student [19, 39] and inpatient [13, 15] samples. In the present sample, Cronbach’s alpha was excellent for the total score (α = .95) as well as the subscales: Nonacceptance of Emotional Responses (α = .92), Difficulties in Engaging in Goal-Directed Behavior (α = .88), Impulse Control Difficulties (α = .88), Lack of Emotional Awareness (α = .84), Limited Access to Emotion Regulation Strategies (α = .91), and Lack of Emotional Clarity (α = .84).

The *Columbia-Suicide Severity Rating Scale* (C-SSRS; [37]), is a clinician-administered rating scale assessing both past and current suicidal ideation and behavior. It measures the constructs of suicide severity, intensity, behavior, and lethality. It has shown excellent internal reliability and a clear and coherent factor structure [28], as well as good convergent/ divergent validity. The instrument developers reported predictive validity to the extent that baseline C-SSRS ratings significantly predicted attempts during treatment with an odds ratio of 1.45 [37]. Cronbach’s alpha for the present sample was .89.

The *WHO Disability Assessment Schedule 2.0* (WHODAS 2.0; [48]) is a 12 item self-report measure that covers the six domains of functioning, including self-care, cognition, life activities, mobility, participation, and getting along with others. Participants are asked to rate how much difficulty they had with each task. Options are rated on a five point Likert type scale ranging from “None” to “Extreme or cannot do.” Example items include “Standing for long periods such as 30 minutes” and “Concentrating on doing something for 10 minutes.” The respondent is then asked to tally the number of days they were unable to carry out their usual activities or had to cut back due to a health condition. The WHODAS 2.0 has exhibited strong validity and reliability [48]. Cronbach’s alpha for the present sample was .89.

The *Structured Clinical Interview for DSM-IV Diagnosis* (SCID-I; [11]; SCID-II; [10]) was administered to all study participants to arrive at standardized, reliable diagnoses. Both the SCID-I, which assesses for Axis I disorders, and the SCID-II, which assesses for Axis II personality disorders, were administered.

### Analytic approach

Differential patterns of emotion dysregulation were examined using latent profile analysis (LPA). LPA is a person-centered modeling technique developed to identify sample subgroups with similar profiles of response [17]. Unique DERS configurations in the current sample were extracted using MPlus 6.1 software [31]. Sequential models specifying between two and five profiles were examined. Final model selection was guided by 1.) considerations involving the functional relation of extracted profiles to various clinical presentations and 2.) global indices of statistical fit [32]. Fit indices included AIC, BIC, and sample size adjusted BIC (aBIC); entropy; and model comparisons on the Lo-Mendell-Rubin (LMR) test. AIC, BIC, and aBIC are standard information criteria wherein lower values indicate incremental improvements in model fit. Entropy, by contrast, is a measure of the degree to which profiles are uniquely characteristic of individual subgroups. Values range from 0 to 1 with entropy ≥ .80 suggesting adequate profile separation [27]. Finally, the LMR contrasts the target model against a solution containing one fewer class. Significant *p*-values indicate improved statistical fit relative to the more parsimonious solution.

Following identification of an acceptable model, emotion dysregulation profiles were examined with respect to a series of external covariates. Differences in suicidal ideation and functional impairment were evaluated for each patient subgroup. Comparisons were examined in SPSS 22.0 using two-tailed tests of statistical significance. Effect sizes for omnibus and pairwise comparisons are reported as η^2^ (small = .010, medium = .060, large = .140) and *d* (small = .20, medium = .50, large = .80), respectively [9].

## Results

### Sample characteristics

DERS scores for the full sample are provided in Table 1. Data approximated a normal distribution (all skew ≤ |.84|, all kurtosis ≤ |1.17|) with no evidence of univariate (z-score ≥ 3.29) or multivariate (Mahalanobis distances *p* < .001) outliers [46]. Comparison with published norms for nonclinical and general inpatient samples indicated elevated dysregulation in this patient group. As expected, scores exceed those observed in untreated student samples, with discrepancies meeting or exceeding conventional standards for large effects across all subscales (*d* = .80 to 1.77; [39]).^1^ Patients with a diagnosis of BPD in the current study also reported greater difficulties relative to general psychiatric inpatients across DERS subscales Nonacceptance (*d* = .48), Goals (*d* = .51), Impulse (*d* = .88), Awareness (*d* = .29), Strategies (*d* = .69), and Clarity (*d* = .36) domains [13, 15].

**Table 1:** Sample and model-implied means for DERS profiles

	Sample (*N* = 156)		Low Impairment (*n* = 41)		Global Dysregulation (*n* = 69)		Emotionally Aware (*n* = 46)	
Scale	*M* ^a^	95% CI	*M* ^b^	95% CI	*M* ^b^	95% CI	*M* ^b^	95% CI
Nonaccept	19.83	[18.75, 20.91]	15.55	[13.22, 17.89]	23.56	[21.92, 25.20]	18.46	[15.42, 21.51]
Goal	19.64	[18.93, 20.35]	14.77	[12.90, 16.65]	21.44	[20.49, 22.38]	21.70	[20.47, 22.93]
Impulse	20.25	[19.28, 21.22]	14.83	[13.02, 16.65]	23.24	[21.43, 25.05]	21.08	[18.83, 23.32]
Awareness	18.44	[17.58, 19.30]	19.82	[18.20, 21.45]	21.24	[19.71, 22.77]	12.94	[10.20, 15.67]
Strategies	28.22	[27.12, 29.33]	20.40	[18.17, 22.62]	32.26	[30.85, 33.67]	29.84	[27.55, 32.13]
Clarity	15.07	[14.38, 15.76]	15.00	[13.65, 16.35]	18.02	[16.62, 19.43]	10.77	[9.11, 12.42]

*Nonaccept* DERS nonacceptance of emotional responses, *Goal* DERS difficulties engaging in goal-directed behavior, *Impulse* DERS impulse control difficulties, *Awareness* DERS lack of emotional awareness, *Strategies* DERS limited access to emotion regulation strategies, *Clarity* DERS lack of emotional clarity

^a^ Sample *SD* for DERS scales are 6.83, 4.50, 6.13, 5.43, 7.00, and 4.38 for Nonaccept, Goal, Impulse, Awareness, Strategies, and Clarity, respectively

^b^ Variance estimates for model implied means are held constant across profiles. Model-implied *SD* for DERS scales are 5.89, 3.30, 5.01, 4.08, 4.87, 3.16 for Nonaccept, Goal, Impulse, Awareness, Strategies, and Clarity, respectively

### Response profiles

Indices of model fit are provided in Table 2. Analyses reached convergence for models specifying between two and four patient classes. AIC, BIC, and aBIC values decreased across each solution. LMR, by contrast, indicated no statistical benefit associated with the extraction of a fourth profile as compared to the 3-class solution. Entropy values suggested adequate separation in the 2- and 3-class models.

**Table 2:** Fit indices for latent profile models at admission (*N* = 156)

Model	AIC	BIC	aBIC	LMR	Entropy
2-Class	5768.3	5826.2	5766.1	0.050	0.809
3-Class	5696.8	5776.1	5693.8	0.018	0.801
4-Class	5663.9	5764.6	5660.1	0.400	0.784

*AIC* akaike information criterion, *BIC* Bayesian information criterion, *aBIC* adjusted Bayesian information criterion, *LMR p*-value for the Lo-Mendell-Rubin

**Fig. 1:**
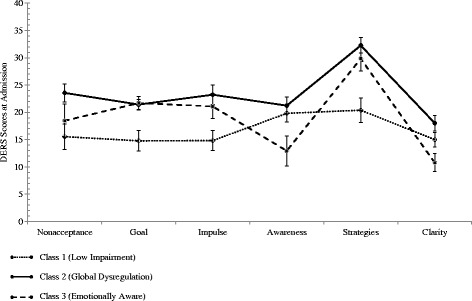
Profile means and 95% confidence intervals for 3-class solution at admission

Given these results, specific profiles were examined for the 3- and 4-class solutions. The first profile of the 3-class solution identified a patient subgroup (28.1%) reporting levels of dysregulation as relatively low compared to the sample as a whole but elevated relative to a nonclinical sample (see Table 2). This group was labelled “Low Impairment” because their reported Difficulties in Engaging in Goal-Directed Behavior interference does not differ from the nonclinical sample. Confidence intervals around model-implied means for this group (see Fig. 1) indicated lower scores for Goal, Impulse, and Strategy subscales relative to other profiles in this solution. The second profile isolated a subgroup of cases (42.9%) reporting elevated levels of dysregulation. Scores in this “Global Dysregulation” group exceeded those estimated for Low Impairment patients on all subscales with the exception of Awareness. Patients in the final profile, dubbed “Emotionally Aware,” (29.0%) evidenced an intermediate pattern of response. Confidence intervals suggest difficulties with goal-directed behavior, impulse control, and access to regulation strategies similar to those reported in the Dysregulated group; however, relative strengths were noted with regard to emotional awareness and clarity. Despite difficulties with the regulation of overt behavior, patients with this profile report an awareness of emotional reactions similar to that observed in non-clinical samples [19, 39].

Low Impairment, Global Dysregulation, and Emotionally Aware profiles were replicated in the 4-class solution along with an additional intermediate group containing moderate to high scores across all DERS subscales. Consistent with the low entropy value for this solution, no domain of functioning was found to uniquely characterize this patient group. As a consequence, the profiles in the 3-class model were selected for further investigation given evidence of adequate fit and greater conceptual clarity.

### Profile comparisons

A series of one-way analysis of variance (ANOVA) models was conducted to determine if there was a significant difference among the three classes with regard to demographics, functional impairment and suicidal ideation at both admission and discharge. Results revealed no significant differences for age [*F*(2153) = 1.28, *p* = .282, η^2^ = 0.01], length of stay [*F*(2153) = .27, *p* = .763, η^2^ = 0.00], number of previous therapists [*F*(2153) = .27, *p* = .760, η^2^ = 0.00], number of previous psychiatrists [*F*(2153) = 1.13, *p* = .325, η^2^ = 0.01], and number of acute hospitalizations [*F*(2153) = 2.36, *p* = .098, η^2^ = 0.03]. There were, however, significant differences in the number of extended psychiatric hospitalizations [*F*(2153) = 3.94, *p* = .021, η^2^ = 0.05] as posthoc tests revealed significantly more extended hospitalizations in the Global Dysregulation group (*M* = 2.29, *SD* = 3.61) than both the Low Impairment (*M* = 1.02, *SD* = 1.81, *p* = .020; *d* = .41) and the Emotionally Aware Groups (*M* = 1.07, *SD* = 1.73, *p* = .021; *d* = .41). Results revealed significant differences among the three groups at admission for suicide ideation intensity (lifetime) [*F*(2153) = 8.05, *p* < .001, η^2^ = 0.10], suicide ideation intensity (within 2 months of admission) [*F*(2153) = 3.39, *p* = .036, η^2^ = 0.04], and functional impairment at admission [*F*(2153) = 6.52, *p* = .002, η^2^ = 0.08]. Tukey post hoc tests revealed that for lifetime suicidal ideation, there were significant differences between the Global Dysregulation group (*M* = 15.10, *SD* = 4.16) and both the Low Impairment (*M* = 11.71, *SD* = 4.95, *p* = .001; *d* = .76) and the Emotionally Aware Groups (*M* = 12.91, *SD* = 4.52, *p* = .030; *d* = .51). Similar results were revealed for suicidal ideation within 2 months of admission, where significant differences were found between the Global Dysregulation group (*M* = 13.69, *SD* = 6.74) and both the Low Impairment (*M* = 10.76, *SD* = 7.66; *p* = .039; *d* = .41) and the Emotionally Aware Groups (*M* = 10.63, *SD* = 7.35; *p* = .026; *d* = .44). With regard to functional impairment, there was a significant difference at admission between the Low Impairment Group (*M* = 16.32, *SD* = 8.11) and the Global Dysregulation group (*M* = 22.72, *SD* = 8.84; *p* = .002; *d* = .75). No significant difference was found between Low Impairment and Emotionally Aware (*M* = 19.17, *SD* = 10.51; *p* = .451, *d* = .30) or between Global Dysregulation and Emotionally Aware groups (*p* = .134, *d* = .37). At discharge, no significant differences were found for suicide ideation intensity in the prior 2 weeks [*F*(2148) = 1.37, *p* = .257, η^2^ = 0.02] or functional impairment [*F*(2148) = 0.03, *p* = .97, η^2^ = 0.00].

### Emotion dysregulation at discharge

As our latent profile analysis was conducted utilizing data collected at hospital admission, a series of ANOVAs were conducted to determine if significant differences between the three groups remained at discharge, after a full treatment course of inpatient hospitalization. Results revealed significant differences between groups for each DERS subscale: nonacceptance [*F*(2148) = 5.20, *p* = .007, η^2^ = 0.07], goals [*F*(2148) = 8.16, *p* < .001, η^2^ = 0.10], impulse [*F*(2148) = 9.07, *p* < .001, η^2^ = 0.11], awareness [*F*(2148) = 8.77, *p* < .001, η^2^ = 0.11], strategies [*F*(2148) = 8.28, *p* < .001, η^2^ = 0.10], and clarity [*F*(2148) = 12.58, *p* < .001, η^2^ = 0.15]. Tukey posthoc tests comparing the Global Dysregulation and the Low Impairment groups revealed the Global Dysregulation group scored higher on each subscale, with significant differences on each subscale *except* the awareness scale (see Table 3 for means). Comparisons between the Low Impairment group and the Emotionally Aware group revealed significant differences on the goals subscale, with the Emotionally Aware group exhibiting higher scores (greater goal interference) at discharge. Finally, posthoc comparisons between the Global Dysregulation group and the Emotionally Aware group revealed significant differences on the impulse, awareness, and clarity subscales, with the Global Dysregulation group reporting higher scores at discharge.

**Table 3:** Discharge means, standard deviations, and 95% confidence intervals for DERS scores

	Low Impairment (*n* = 38)		Global Dysregulation (*n* = 67)		Emotionally Aware (*n* = 46)	
Scale	*M(SD)*	95% CI	*M(SD)*	95% CI	*M(SD)*	95% CI
Nonaccept	11.74 (4.75)^a^	[10.23, 13.24]	15.58 (6.51)^b^	[13.99, 17.17]	13.11 (6.77)^ab^	[11.10, 15.12]
Goal	12.00 (4.43)^a^	[10.54, 13.46]	15.97 (5.23)^b^	[14.70, 17.24]	15.46 (5.14)^b^	[13.93, 16.98]
Impulse	11.05 (4.32)^a^	[9.63, 12.47]	15.45 (6.02)^b^	[13.98, 16.92]	12.72 (4.89^a^	[11.26, 14.17]
Awareness	15.55 (5.29)^ab^	[13.81, 17.29]	17.24 (5.29)^a^	[15.95, 18.53]	13.11 (4.79)^b^	[11.68, 14.53]
Strategies	15.61 (6.01)^a^	[13.61, 17.60]	21.82 (8.07)^b^	[19.84, 23.77]	19.24 (7.74)^ab^	[16.94, 21.54]
Clarity	11.89 (4.35)^a^	[10.47, 13.32]	13.85 (4.20)^b^	[12.83, 14.88]	10.77 (3.63)^a^	[9.96, 11.03]

*Nonaccept* DERS nonacceptance of emotional responses, *Goal* DERS difficulties engaging in goal-directed behavior, *Impulse* DERS impulse control difficulties, *Awareness* DERS lack of emotional awareness, *Strategies* DERS limited access to emotion regulation strategies, *Clarity* DERS lack of emotional clarity. Means with the same superscripts within a row are not significantly different. Means with different superscripts within a row are significantly different at the .05 level, as determined by Tukey’s post hoc test

## Discussion

The present results are consistent with extant research highlighting emotion dysregulation as a key feature of BPD [8, 23]. Beyond this, the present results indicate important differences in type and degree of emotion dysregulation among individuals with BPD. Profiles were distinguished by differences across a range of emotion regulation features, including awareness and clarity regarding emotions, perceived capacity to manage emotional distress, and the extent to which dysregulated emotions interfere with daily functioning and goal achievement. It should be noted here that the designation of patterns as Emotionally Aware and Low Impairment is not meant to suggest an absence of psychopathology in patients manifesting these patterns, in that all participants in the study were psychiatrically hospitalized and met diagnostic criteria for BPD. Indeed, it is one of the notable findings of this study that some patients diagnosed with BPD failed to exhibit impairing levels of emotional dysregulation in specific domains.

The Global Dysregulation group, consisting of 48% of the sample, most closely approximates the profile of an individual with the affective subtype described by Oldham [35], characterized by a pronounced deficit in emotional regulation skills, poor acceptance of emotions, impulsivity related to emotional distress, and significant interference of emotions with goal achievement and functioning. This group evidenced more severe suicidal ideation and impairment in global functioning, relative to the other two groups.

The Emotionally Aware group (29%) was notable for its self-reported attention to and understanding of emotions. However, this quality apparently does not translate into functional capacity, as this group strongly resembles the Global Dysregulation group in terms of low confidence in emotional regulation strategies and interference of emotional distress with goal achievement. Nevertheless, this group reported lower levels of suicidal ideation and global impairment compared to the Global Dysregulation group. Reasons for this apparent split between dysregulation and suicidal ideation await further study.

The Low Impairment group (28%) is notable for its lack of self-reported interference of emotions with functioning and goal achievement, with a mean score on the Difficulties in Engaging in Goal-Directed Behavior scale roughly equivalent to that reported for a nonclinical sample [19]. Although they indicate relatively low concern about emotional awareness and clarity, this profile leaves one hard-pressed to conclude that emotional dysregulation is a clinically serious concern for this group of BPD patients. This raises the interesting prospect of a sizable subgroup of patients who meet BPD diagnostic criteria, but with relatively low levels of emotional dysregulation. This is certainly a diagnostic possibility, given the other possible combinations of diagnostic criteria that would justify the diagnosis; however, it is not a symptom profile that comports well with the view of emotional dysregulation as a “core feature” of BPD.

This study does not provide conclusive answers to the question of whether the profile patterns described here might be applied clinically toward more specific treatment planning. The robustness of the classification is underscored by the fact that, rather remarkably, the relative rankings of the three groups on all DERS subscales remained intact at post-treatment; for example, the Global Dysregulation group continued to score highest (most impaired) on Strategies, with the Low Impairment group scoring lowest. Supplemental analyses of DERS scores at discharge provide additional evidence for the relative stability of extracted profiles (see Additional file 1).^2^

**Fig. 2:**
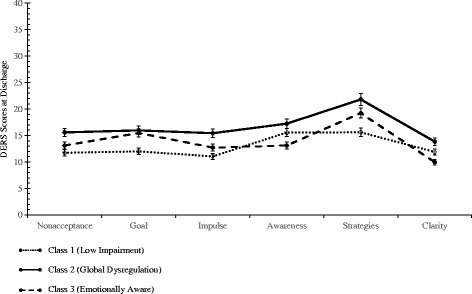
Means and 95% confidence intervals for 3-class solution at discharge

On the other hand, these results are remarkable for the finding that, notwithstanding their various pre-treatment differences, decreases in DERS scores were noted over the course of hospitalization (Fig. 2) with supplemental LPA at discharge providing evidence of transitions to less severe profiles (see Additional file 1). Consistent with this pattern, at discharge, the three groups’ differences in suicidal ideation and global functioning were no longer statistically significant. LPA-informed classification allowed for the examination of between-group differences on measures of suicidality and functional impairment at pre-treatment, admission, and discharge. One class of patients (Global Dysregulation) reported significantly higher levels of lifetime suicidality relative to members of the two other classes. The same class of patients also reported a significantly higher level of functional impairment relative to members of one other class at admission. The fact that the Dysregulated group comes to resemble the other two groups over the course of hospitalization indicates significant therapeutic response to treatment, a result consistent with previous findings with the broader inpatient population showing major improvement in emotion dysregulation over the course of 6 to 8 weeks of hospitalization [13, 15, 16]. However, while all groups showed improvement, all groups failed to normalize, leaving the most impaired groups room for further improvement. Due to the stability in the relative rankings of the groups, it is hoped that individualized treatment targeting the specific needs of each group will further accelerate their treatment course.

Several limitations of this study should be noted. First this group of participants reported significantly higher educational attainment and income and was racially and ethnically less diverse than most psychiatric populations. Replication with more diverse groups will be needed before these results can be considered generalizable. Second, although participants in all three profile groups showed improvement over the course of hospitalization, it is not possible to identify what therapeutic factors they responded to, or even whether the three groups all were responding to the same therapeutic influences. Thus, specific guidance regarding optimal, specific interventions for these three groups must await studies with controls for such influences.

Finally, we should note that this profile analysis is not intended as a subtyping system for borderline personality disorder. However, future research can provide additional ways of examining this issue. For example, other theories of BPD and emotion dysregulation, such as the one discussed by Carpenter and Trull [7] are in need of empirical study. Furthermore, although some have suggested BPD subtypes (e.g., [30]), a wide assortment of factors in addition to emotional dysregulation would need to be taken into account. Individuals with BPD differ widely in terms of both symptoms (such as suicidality, nonsuicidal self-injury, and substance abuse) and learning histories (such as trauma experiences, whether in childhood or adulthood). It is to be hoped that the results reported here will be useful in ultimately better understanding and developing more effective treatments for individuals with borderline personality disorder in its various forms.

## Conclusions

Results revealed that a three class solution best fit the sample (Low Impairment, Global Dysregulation, and Emotionally Aware) and that at admission, the Global Dysregulation group reported significantly higher suicidal ideation than either the Low Impairment or Emotionally Aware groups, and that the Global Dysregulation group reported significantly higher functional impairment than the Low Impairment group. Although all three groups improved greatly over the course of hospital treatment, they remained distinguishable at discharge, retaining their positions symptomatically relative to one another. The results of the present study add to the literature in an attempt to better understand BPD and provide more effective individualized treatment.

## Additional file

Additional file 1: Figures showing repeat LPAs at admission and discharge for each group. (DOCX 23 kb)

## Abbreviations

aBIC: Adjusted Bayesian information criterion
AIC: Akaike information criterion
ANOVA: Analysis of variance
BIC: Bayesian information criterion
BPD: Borderline personality disorder
C-SSRS: Columbia-suicide severity rating scale
DBT: Dialectical behavior therapy
DERS: Difficulties in emotion regulation scale
DSM: Diagnostic and statistical manual
LMR: Lo-Mendell-Rubin
LPA: Latent profile analysis
SCID-I/II: Structured clinical interview for DSM-IV disorders
SSDI: Social security disability income
WHODAS 2.0: WHO Disability assessment schedule 2.0

## Footnotes

1 Effect size estimates were calculated using means and standard deviations from Salsman & Linehan’s [39] female sample.

2 The authors would like to thank the anonymous reviewer for the suggestion to repeat the LPA at discharge.
