# Supplementary material for: Variations of emotion dysregulation in borderline personality disorder: a latent profile analysis approach with adult psychiatric inpatients
Source: Borderline Personal Disord Emot Dysregul. 2017 Aug 24;4:17. doi: 10.1186/s40479-017-0068-2 (PMC5569486; doi:10.1186/s40479-017-0068-2)
Supplement: Additional file 1: — Figures showing repeat LPAs at admission and discharge for each group. (DOCX 23 kb) [file 40479_2017_68_MOESM1_ESM.docx]

Appendix A.

Figures showing repeat LPAs at admission and discharge for each group.

DERS scores at admission and discharge for Low Impairment Groups

DERS scores at admission and discharge for Global Dysregulation Groups

DERS scores at admission and discharge for Emotionally Aware Groups
